# Outcomes of Fibular Nailing: Experience from a Single Center

**DOI:** 10.7759/cureus.85408

**Published:** 2025-06-05

**Authors:** Chun Liang Lau, Kwong-Lee Wan, Kit Chuen Wong, Yee Tong Chong, Gurmeet Singh Sewa Singh

**Affiliations:** 1 Orthopaedics/Foot and Ankle Surgery, Hospital Seberang Jaya, Penang, MYS

**Keywords:** fibula fracture, fibular nail, foot and ankle fracture, rod, weber

## Abstract

This case series reports on the outcomes of eight patients with Weber B and Weber C fibular fractures, with or without associated tibial fractures, treated using the Trauhui fibular nail (Trauhui Medical, Jiangsu, China), the only fibular intramedullary nail currently available in Malaysia. All procedures were performed by a single surgeon at a single center between April and October 2024. Clinical and radiological union was achieved in seven of the eight patients, with a mean union time of 10 weeks. One patient developed a nonunion. Additionally, one case of superficial surgical site infection was observed. At the six-month follow-up, functional outcomes were favorable, with a mean American Orthopaedic Foot & Ankle Society (AOFAS) score of 85 and a mean Visual Analog Scale (VAS) score of one. These findings suggest that the fibular nail is a generally safe and effective treatment option for Weber B and C fibular fractures.

## Introduction

Intramedullary fixation of fibula fractures with fibular nails has seen a resurgence in interest, although comprehensive outcome data remains limited [1]. Modern fibular nails offer potential advantages, including syndesmosis fixation via distal locking screws [2]. The appeal of intramedullary fixation lies in its minimally invasive nature, potentially leading to fewer wound complications compared to traditional plate fixation, especially in high-risk pilon fractures [3]. The purpose of this study is to evaluate the outcomes, complication rates, and implant removal rates associated with fibula nail fixation utilizing both proximal and distal fixation capabilities performed by a single experienced surgeon. This case series aims to contribute to the existing literature by presenting a detailed analysis of clinical and radiological outcomes in a cohort of patients treated with fibular nailing at a single center. Intramedullary implants have a history of success in treating long bone fractures, and recent studies have explored various intramedullary implants for fibular fracture treatment [4]. This study will add depth to the understanding of fibular nail fixation in a defined patient population, with a focus on surgical technique, fracture characteristics, and postoperative rehabilitation protocols, with the intent to provide practical insights for orthopedic surgeons considering this fixation method.

## Materials and methods

This retrospective case series was conducted at Hospital Seberang Jaya, Penang, Malaysia, and analyzed data were collected from eight consecutive patients who underwent fibular nailing for unstable ankle fractures at a single institution between April 2024 and December 2024. Patient demographics, fracture characteristics, surgical technique, complications, and radiographic and clinical outcomes were recorded. Inclusion criteria consisted of skeletally mature patients presenting with acute, unstable ankle fractures involving the fibula who were treated with intramedullary nail fixation. Exclusion criteria included open fractures at initial presentation (though these may be considered for inclusion post debridement), pathological fractures, and patients lost to follow-up. The fractures were classified according to the Weber classification system. All surgeries were performed by a single, experienced orthopedic surgeon specializing in foot and ankle surgery, utilizing a standardized surgical technique. Data collected included patient age, sex, body mass index (BMI), medical comorbidities (e.g., diabetes, peripheral vascular disease), fracture type, time to surgery, operative time, length of hospital stay, complications (both intraoperative and postoperative), need for secondary procedures, and radiographic evidence of union. Patients were considered high risk if they had medical comorbidities at the time of injury that have been shown in current literature to increase surgical complication risk [5].

**Figure 1:**
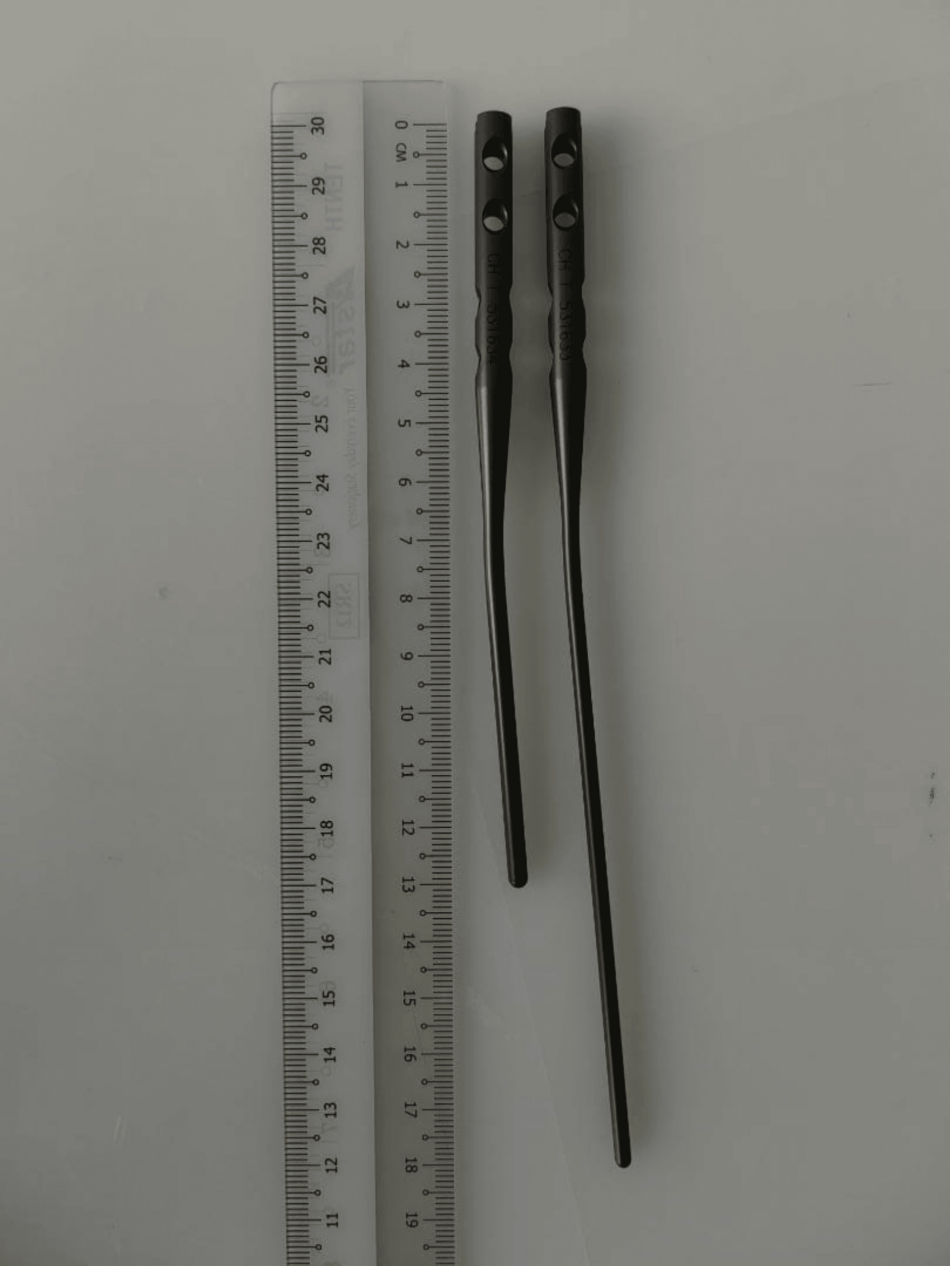
Trauhui fibular nail (Trauhui Medical, Jiangsu, China) This figure is original and derived from data collected in the present study.

The fibular nail implant used in the surgery was supplied by Trauhui Medical (Jiangsu, China), currently the only available fibular nail system in Malaysia (Figure 1).

This implant is offered in just two lengths, 130 mm and 180 mm, and two diameters: 3.0 mm and 3.6 mm. Distal reamer sizes available for this system include 2.7 mm, 3.1 mm, 3.7 mm, and 4.1 mm. Notably, this nail differs from other, more established fibular nail systems on the market, such as those from Arthrex (Naples, FL), Paragon 28 (Englewood, CO), and Acumed (Hillsboro, OR), in that it lacks proximal locking fixation. While other systems often feature proximal fixation methods, either with locking screws or integrated talon mechanisms, the Trauhui nail does not offer any form of proximal stabilization. This limitation in patient selection for our case series is due to the absence of a proximal locking mechanism, as our primary aim was to achieve length and stability of the fibula.

**Figure 2:**
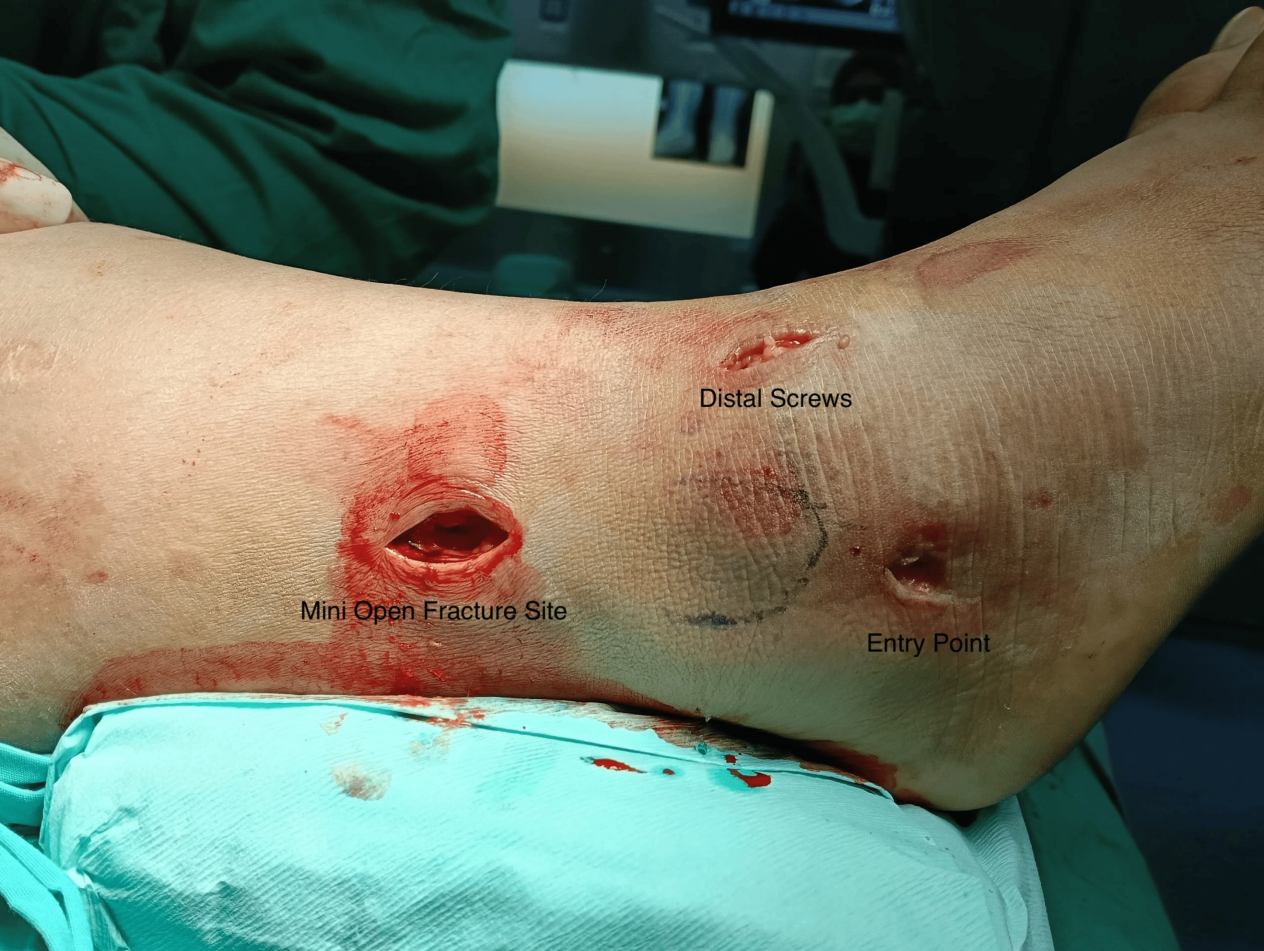
Incisions of a fibular nail This figure is original and derived from data collected in the present study.

The patient is positioned either supine or prone, depending on the presence of any associated posterior malleolus fractures. A small 1 cm vertical incision is made approximately 1 cm distal to the tip of the lateral malleolus (Figure 2).

**Figure 3:**
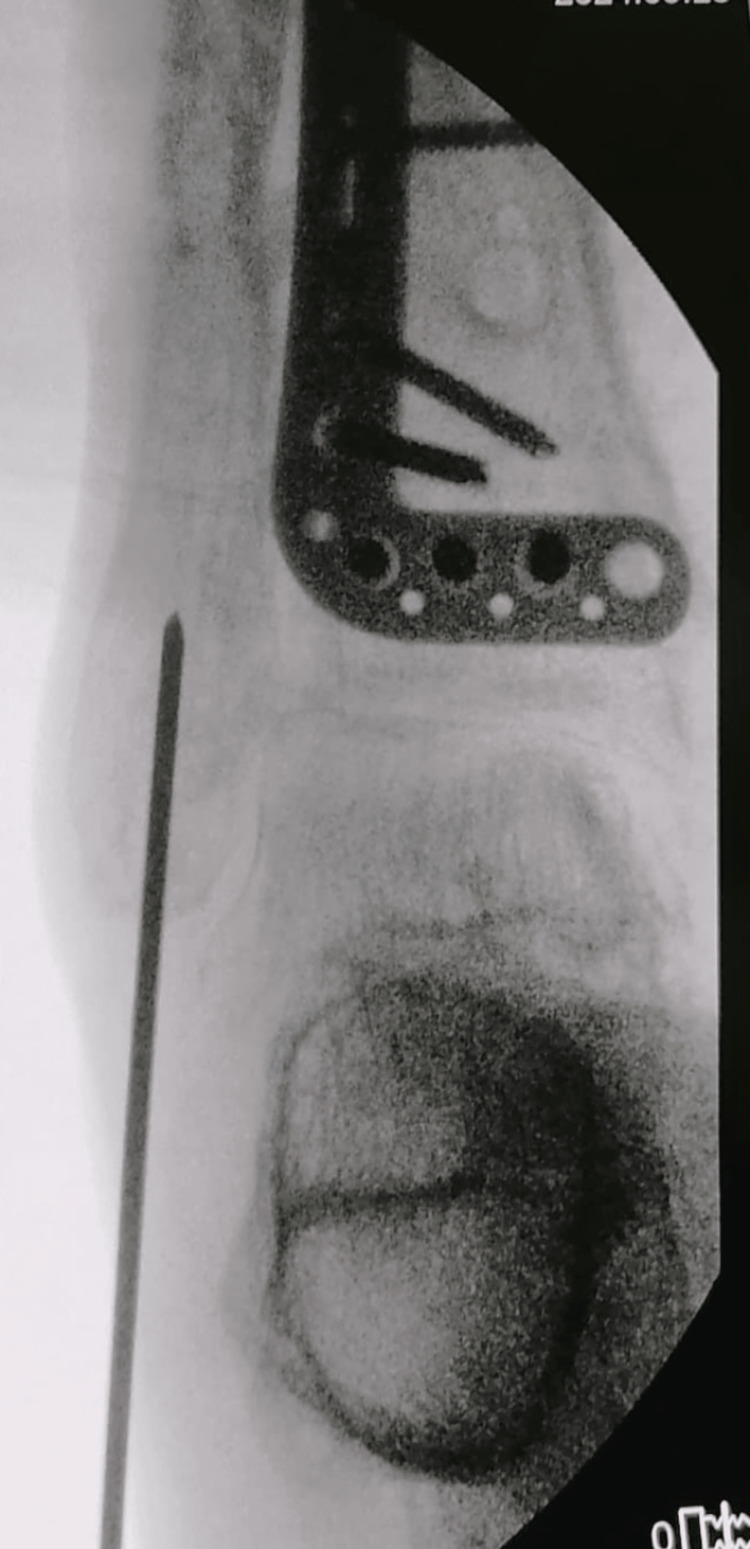
Entry guidewire over the tip of lateral malleolus, aiming to the center of canal This figure is original and derived from data collected in the present study.

Under fluoroscopic guidance, a guide wire is inserted with the entry point centered or slightly medial at the tip of the lateral malleolus (Figure 3).

**Figure 4:**
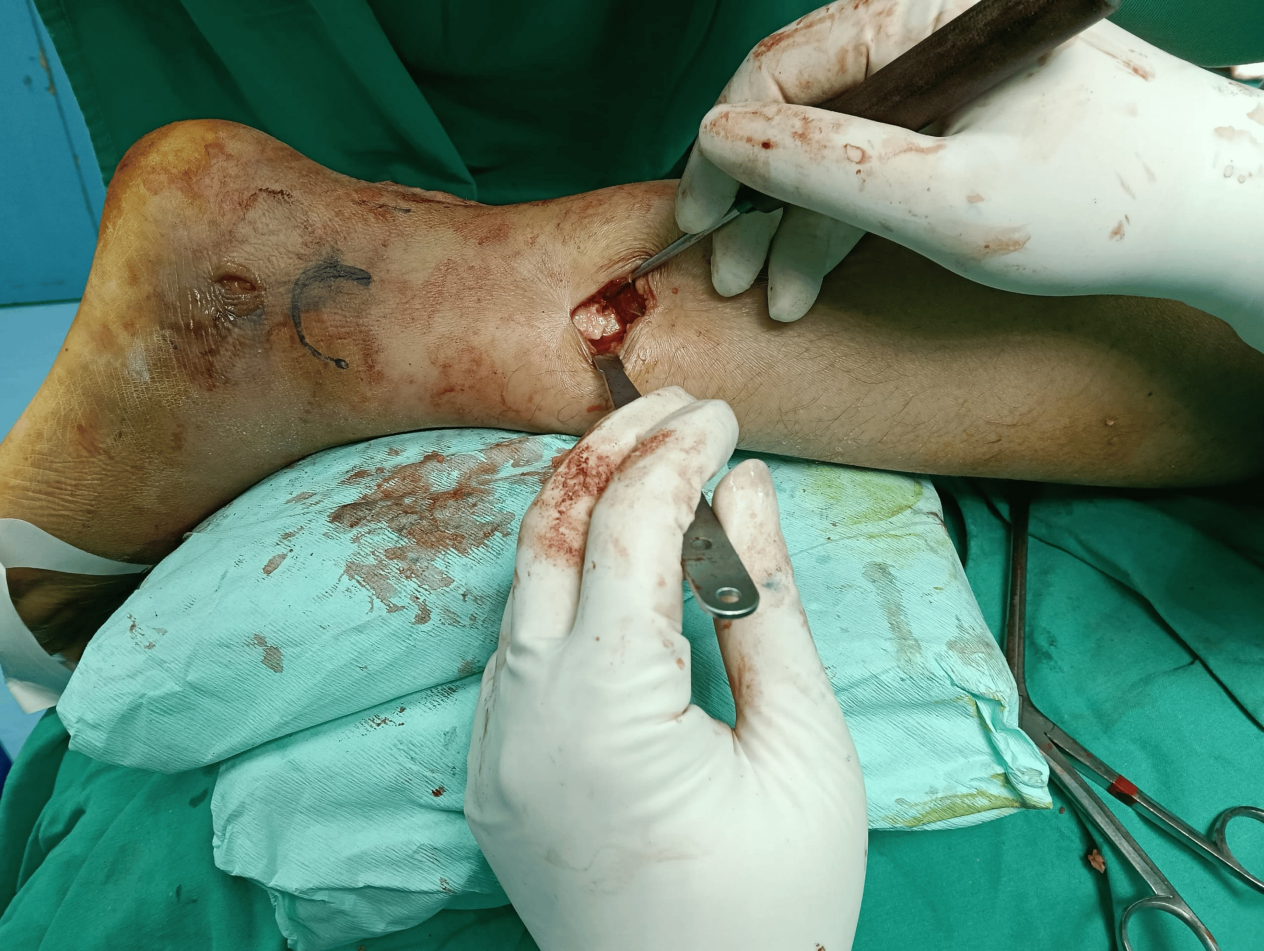
Mini open incision to get an anatomical reduction of the fibular This figure is original and derived from data collected in the present study.

The trajectory is aimed toward the center of the fibular canal in both the anteroposterior (AP) and lateral views. Once satisfactory positioning of the guide wire is confirmed via fluoroscopy, an entry reamer is used to prepare the canal at the entry point. A mini-open incision approximately 2-3 cm in length is made directly over the fracture site to allow for indirect fracture reduction. Kern bone-holding forceps or bone levers are used to achieve anatomical end-to-end alignment of the fibula (Figure 4).

**Figure 5:**
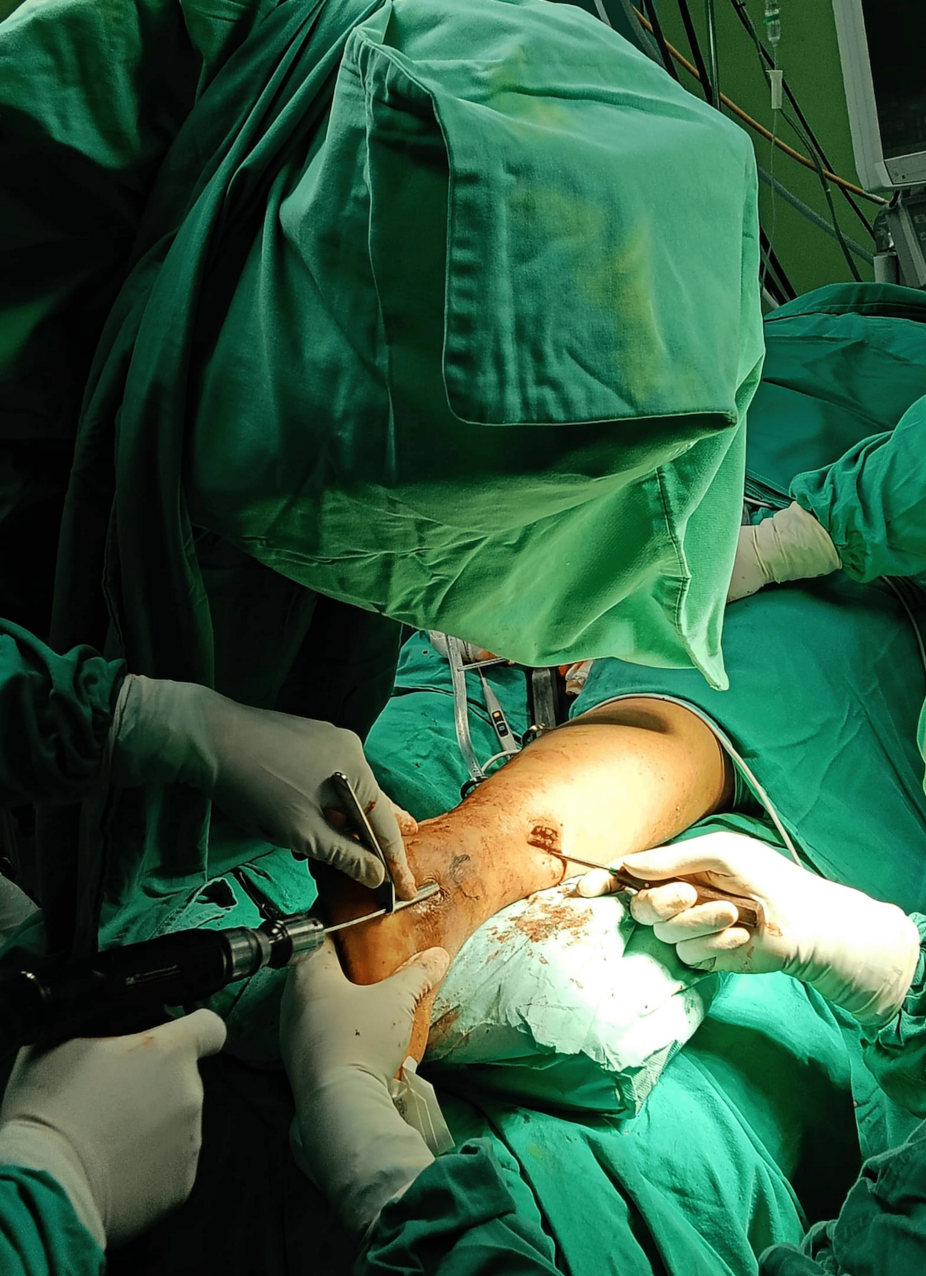
An assistant maintaining the fibular alignment during reaming process This figure is original and derived from data collected in the present study.

Once reduction is confirmed, canal reaming is initiated and continued until the desired diameter is reached. Throughout this process, an assistant maintains the reduction using the instruments to ensure alignment is preserved, which is particularly crucial given that the Trauhui fibular nail lacks proximal locking fixation (Figure 5).

**Figure 6:**
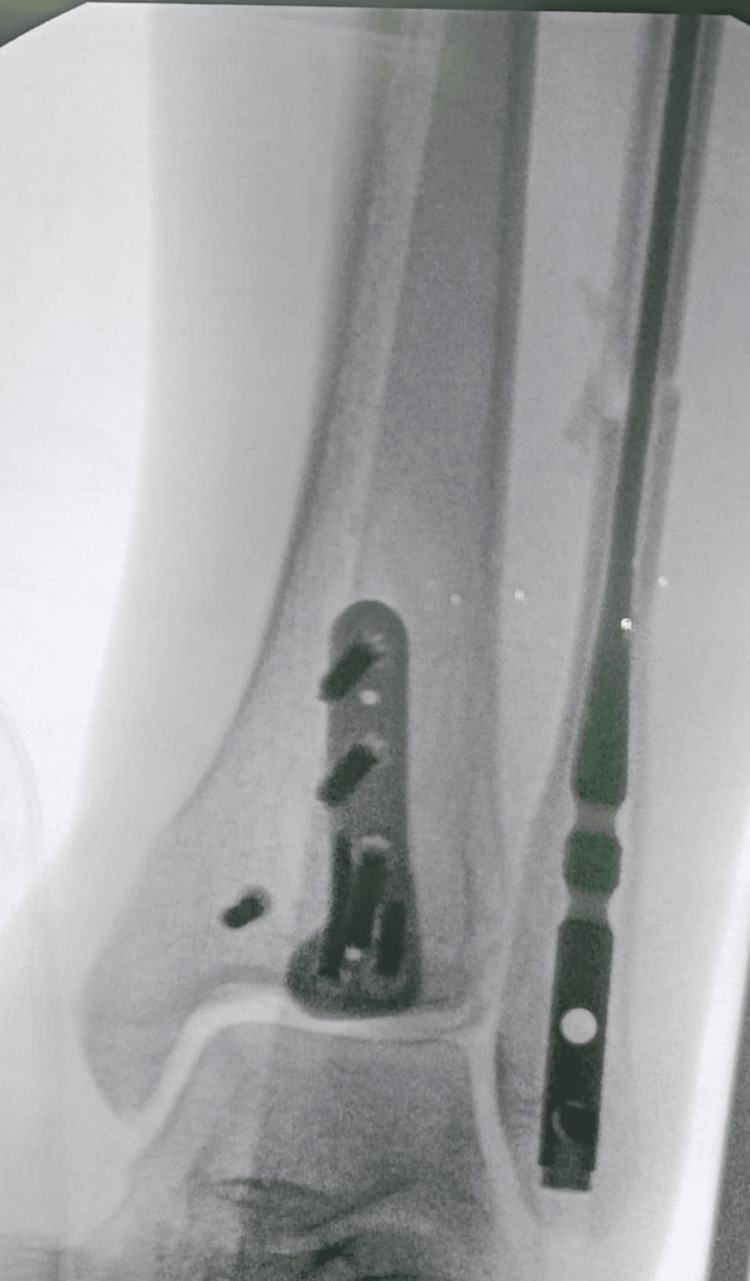
Intraoperative fibular nail fluoroscopy This figure is original and derived from data collected in the present study.

Following reaming, the fibular nail is inserted, ensuring it slides smoothly into the canal without disrupting the fracture alignment. Distal fixation is achieved with one or two locking screws inserted through the provided jig (Figure 6).

Additionally, up to two syndesmotic screws or flexible fixation devices may be placed using the same jig system, consistent with techniques used in other established fibular nail systems.

Postoperatively, patients followed a standard rehabilitation protocol, with weight-bearing as tolerated at six weeks, depending on radiographic evidence of healing. Radiographic assessment of fracture union was performed at regular intervals, with union defined as bridging callus formation on at least three of four cortices on orthogonal radiographs. Clinical outcomes were assessed using the American Orthopaedic Foot & Ankle Society (AOFAS) ankle-hindfoot scale, Visual Analog Scale (VAS) for pain, and range of motion measurements at each follow-up visit. Descriptive statistics were used to analyze the data. 

## Results

**Figure 7:**
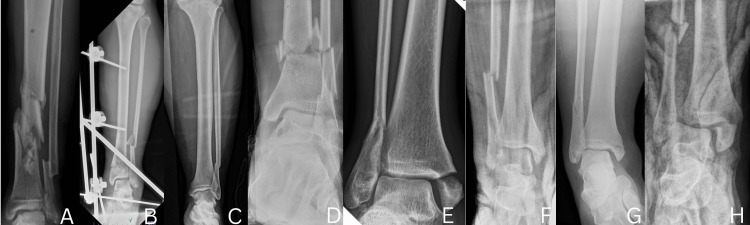
Preoperative radiographs of subjects with various types of ankle fractures treated using fibular nail fixation This figure is original and derived from data collected in the present study. The alphabetical identifiers (e.g., A, B, C, etc.) used in the figure are arbitrary and were created solely for the purpose of referencing specific cases within this article. These identifiers do not correspond to any patient-identifying information.

**Figure 8:**
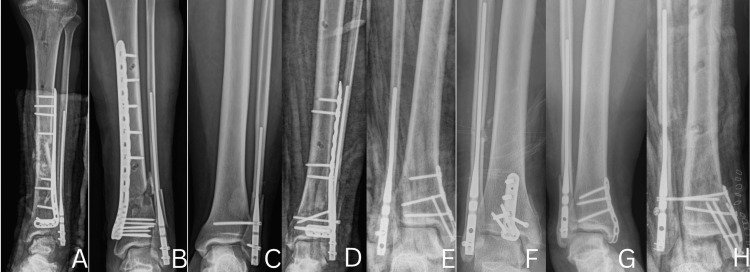
Immediate postoperative radiographs of patients following fibular nail fixation This figure is original and derived from data collected in the present study. The alphabetical identifiers (e.g., A, B, C, etc.) used in the figure are arbitrary and were created solely for the purpose of referencing specific cases within this article. These identifiers do not correspond to any patient-identifying information.

The study initially identified 10 patients; however, two were excluded, one due to loss to follow-up and another who passed away from unrelated medical conditions during the follow-up period, resulting in a final cohort of eight patients included in the analysis, with a mean age of 43 years (range, 23-72 years). The average BMI was 29 kg/m², and 37.5% of patients were current or former smokers. The average follow-up time was nine months (range, six to 12 months). The cohort included four male and four female patients. Common comorbidities included diabetes (n=1), hypertension (n=1), and obesity (n=2). The fractures were classified as Weber B (n=1) and Weber C (n=7). Representative preoperative radiographs (Figure 7) demonstrate the typical fracture patterns observed in this case series. The time to surgery from the date of injury ranged from one to two weeks on average, though in some cases, particularly with open fractures or due to prolonged waiting lists in government healthcare facilities, surgery was delayed up to two months. The average operative time was 29 minutes, measured from the start to the completion of the fibular nail procedure. Immediate postoperative radiographs (Figure 8) illustrate restoration of alignment and implant positioning following fibular nail fixation. The average length of hospital stay was two days.

Fracture union was achieved in all patients at an average of 10 weeks postoperatively. The calculated infection rate for fibular nailing was 12.5%. One patient developed a superficial wound infection, which was successfully treated with oral antibiotics and regular dressings. The nonunion rate was 12.5%; however, the affected patient reported no pain at the fracture site and had already resumed ambulation. One patient exhibited implant breakage over the medial malleolus on follow-up radiographs. Despite this, the fibular fixation remained intact, with complete radiographic union achieved. The patient was ambulating independently without any assistive devices and reported no functional limitations. No cases of malunion were observed in this series, likely due to the consistent use of mini-open reduction techniques in all procedures.

Regarding postoperative management, for cases involving only fibula fractures, partial weight bearing was usually started at six weeks postoperatively, followed by progression to full weight bearing at 10 to 12 weeks. This protocol was followed to optimize healing while minimizing undue stress on the fixation.

One patient initially selected for fibular nailing experienced a hardware-related complication during the procedure; the fibular canal was too narrow, and the fracture extended proximally during nail insertion. The case was converted to open reduction and plating of the lateral malleolus, and this patient was excluded from the final analysis.

At final follow-up, the mean AOFAS score was 85, and the mean VAS score for pain was one (Table 1).

**Table 1:** Patients’ demographic data, fracture characteristics, types of syndesmotic fixation, and six-month outcomes in patients with malleolar ankle fractures treated with fibular nail AOFAS: American Orthopaedic Foot & Ankle Society; VAS: Visual Analog Scale The alphabetical identifiers (e.g., A, B, C, etc.) used in the table are arbitrary and were created solely for the purpose of referencing specific cases within this article. These identifiers do not correspond to any patient-identifying information.

Subject	Age	Gender	BMI	Comorbidity/Smoker	Open/Closed fracture	Types of malleolar fractures/Weber Classification	Syndesmosis involvement /Types of fixation	Radiological union time (weeks)	AOFAS score at six months	VAS score at six months	Complications	Surgery time (Minutes)
A	65	Male	26.1	Smoker	Open	Bimalleolar/Weber C	No	12	87	0	None	32
B	51	Female	29.6	None	Closed	Bimalleolar/Weber C	Yes/Screw Fix	11	94	0	None	28
C	24	Male	26.5	Smoker	Open	Lateral Malleolus/Weber C	Yes/Screw Fix	9	100	0	None	30
D	57	Female	27.8	None	Open	Bimalleolar/Weber C	No	9	86	1	None	32
E	72	Male	26.7	Hypertension	Closed	Bimalleolar/Weber B	No	9	80	1	Broken medial malleolus plate and screw	27
F	26	Female	32.5	Obesity	Closed	Bimalleolar/Weber C	No	9	82	1	None	25
G	23	Female	34.2	Obesity	Closed	Bimalleolar/Weber C	No	11	70	3	Superficial surgical site infection	28
H	24	Male	28.3	Smoker	Open	Bimalleolar/Weber C	Yes/Screw fix + Flexible fixation	10	78	2	Nonunion	27
Mean age	43	Mean BMI	29	Mean radiological union time, AOFAS score, and VAS score				10	85	1	Mean surgery time	29

**Figure 9:**
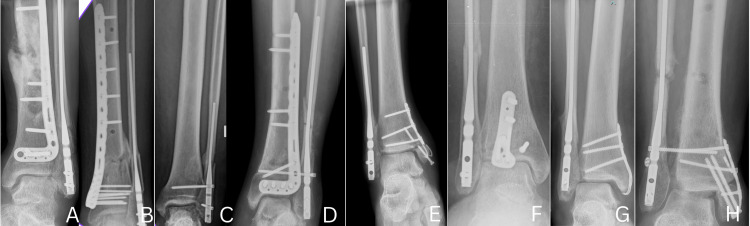
Postoperative radiographs at four to six months demonstrating maintained reduction and radiographic union except for one case of asymptomatic nonunion following fibular nail fixation This figure is original and derived from data collected in the present study. The alphabetical identifiers (e.g., A, B, C, etc.) used in the figure are arbitrary and were created solely for the purpose of referencing specific cases within this article. These identifiers do not correspond to any patient-identifying information.

The average ankle range of motion was within 10 degrees of the contralateral ankle. Analysis of radiographic data revealed no significant loss of reduction or hardware failure in any of the patients (Figure 9). This data corroborate previous findings that intramedullary screw fixation can be successful in most patients [6]. Additionally, our experience supports existing literature indicating that the learning curve for fibular nail fixation is relatively short, with reproducible outcomes achievable after a limited number of cases [7]. Despite the favorable outcomes, whether fibular nail insertion causes damage to the lateral ankle ligaments, specifically the anterior talofibular ligament (ATFL) and calcaneofibular ligament (CFL), remains uncertain. However, talar tilt and anterior drawer tests performed at six months postoperatively were negative in all patients, suggesting no clinical evidence of lateral ligament injury.

## Discussion

This single-center case series provides preliminary evidence that fibular nailing can be an effective treatment option for unstable ankle fractures, with acceptable union rates and functional outcomes. Fibular nails offer a reliable alternative fixation technique in the subset of patients who have significant soft tissue injuries [8]. The minimally invasive nature of the procedure may reduce the risk of wound complications compared to traditional open reduction and internal fixation, particularly in patients with compromised soft tissues or medical comorbidities [9]. Several studies have advocated for the use of fibular nails in ankle fractures, even in patients at high risk for wound complications [10].

The findings of this study should be interpreted with caution, as it represents a single-center experience with a limited number of patients. A larger, multi-center study with a control group would provide more definitive evidence regarding the effectiveness and safety of fibular nailing compared to other fixation methods. Additionally, longer-term follow-up is needed to assess the durability of the fixation and the potential for late complications. 

Furthermore, the utilization of intramedullary nails has demonstrated simplicity and reproducibility, facilitating early weight-bearing and potentially accelerating fracture healing [11]. Moreover, the closed reduction and indirect fixation techniques associated with intramedullary nails minimize disruption of the surrounding soft tissues, thereby preserving vascular supply and reducing the risk of infection and nonunion [12]. The nail acts as an internal splint, providing stability and maintaining alignment while minimizing stress on the fracture site. The intramedullary approach preserves the soft tissue envelope, potentially reducing the risk of wound complications [13]. The biomechanical stability afforded by intramedullary fixation promotes early weight-bearing, which is known to stimulate bone healing and improve functional outcomes [14]. 

The current literature supports the use of intramedullary fixation in fibular fractures, particularly in cases with poor soft tissue or significant comorbidities [15]. Intramedullary implants have a history of success in long bone fractures. There is evidence that intramedullary fixation of fibular fractures reports significantly lower rates of wound complications. The technique is minimally invasive and allows for earlier operative intervention in instances where traditional approaches might be delayed to allow for the improvement of soft tissue swelling. The reduced exposure translates to less soft tissue dissection, diminished hardware prominence, and decreased periosteal stripping [16]. 

The use of intramedullary nails in the fibula is an evolving technique that has demonstrated potential benefits compared to traditional plating techniques. Intramedullary fixation facilitates load sharing, where the implant and bone share compressive, bending, and torsional loads, thus promoting fracture healing. The advent of specialized instrumentation and locking screw designs has enhanced the stability and versatility of fibular nails, allowing for their use in a wider range of fracture patterns. Intramedullary fixation allows for indirect reduction techniques, minimizing periosteal stripping and soft tissue trauma, which can be particularly advantageous in fractures with associated soft tissue injuries. Intramedullary fixation of fibular fractures was first described utilizing the Inyo Nail (Orthopedic Designs North America, Incline Village, NV) in 1986. More contemporary designs allow for increased fracture stability with multiple points of fixation proximally and distally.

It has been shown that complication rates vary with different implants but are generally low. Newer generation intramedullary nails are now available, but more research is needed to determine the optimal fixation strategy for different fracture patterns and patient populations [17]. Surgeons should be aware of the potential complications associated with fibular nailing, such as malreduction, nonunion, hardware failure, and infection, and take appropriate measures to prevent and manage these issues. While this case series provides valuable insights into the outcomes of fibular nailing, further research is needed to validate these findings and optimize the technique for various fracture patterns and patient populations.

Study limitations

Although this case series demonstrates encouraging outcomes, several limitations must be acknowledged. The small sample size and retrospective design limit the generalizability of the findings. Moreover, a formal sample size calculation was not performed, as this study was designed as an observational case series. Additionally, the absence of a control group prevents direct comparison of fibular nailing outcomes with those of conventional fixation methods, such as plate osteosynthesis [18]. Another limitation is that the study did not include more complex fracture types, such as comminuted fractures, which may influence the applicability of the findings to more severe injury patterns. Furthermore, the study did not demonstrate a significant difference in postoperative weight-bearing status when compared to plate fixation, limiting conclusions about functional advantages in that aspect.

Despite these limitations, fibular nailing appears to offer distinct advantages in selected patients, particularly those with compromised soft tissue envelopes, due to its minimally invasive nature. The technique requires only small incisions for nail insertion and locking screw placement, thereby reducing soft tissue disruption and preserving periosteal blood supply. These characteristics make it a potentially favorable option in patients at higher risk of wound complications, such as those with diabetes, prior surgery, or peripheral vascular disease.

## Conclusions

Fibular nailing has emerged as a promising alternative to traditional plate fixation for unstable ankle fractures, especially in patients with compromised soft tissues or significant medical comorbidities. It is associated with lower wound complication rates, high union rates, and satisfactory functional outcomes, particularly in cases involving distal tibia fractures. Intramedullary fixation offers advantages such as minimally invasive techniques, reduced operative time, and decreased soft tissue disruption, which may lower the risk of infection. However, current evidence is limited, with a lack of high-quality comparative studies. Further research, including biomechanical analyses, long-term outcome evaluations, and cost-effectiveness studies, is needed to establish the definitive role of fibular nailing in managing ankle fractures and to refine surgical techniques and implant designs.
